# Information fusion control with time delay for smooth pursuit eye movement

**DOI:** 10.14814/phy2.12775

**Published:** 2016-05-26

**Authors:** Menghua Zhang, Xin Ma, Bin Qin, Guangmao Wang, Yanan Guo, Zhigang Xu, Yafang Wang, Yibin Li

**Affiliations:** ^1^ School of Control Science and EngineeringShandong UniversityJinanChina; ^2^ School of Life ScienceShandong UniversityJinanChina; ^3^ School of Computer Science and TechnologyShandong UniversityJinanChina

**Keywords:** Eye movement, information fusion control, learning, prediction, smooth pursuit, time delays

## Abstract

Smooth pursuit eye movement depends on prediction and learning, and is subject to time delays in the visual pathways. In this paper, an information fusion control method with time delay is presented, implementing smooth pursuit eye movement with prediction and learning as well as solving the problem of time delays in the visual pathways. By fusing the soft constraint information of the target trajectory of eyes and the ideal control strategy, and the hard constraint information of the eye system state equation and the output equation, optimal estimations of the co‐state sequence and the control variable are obtained. The proposed control method can track not only constant velocity, sinusoidal target motion, but also arbitrary moving targets. Moreover, the absolute value of the retinal slip reaches steady state after 0.1 sec. Information fusion control method elegantly describes in a function manner how the brain may deal with arbitrary target velocities, how it implements the smooth pursuit eye movement with prediction, learning, and time delays. These two principles allowed us to accurately describe visually guided, predictive and learning smooth pursuit dynamics observed in a wide variety of tasks within a single theoretical framework. The tracking control performance of the proposed information fusion control with time delays is verified by numerical simulation results.

## Introduction

Primates have to move their eyes to acquire accurate information about small moving targets due to their narrow foveal vision (Shibata et al. 2005). Smooth pursuit eye movements ensure that image velocity stays within a range that is best for visual acuity and visibility (Kowler 2011; Adams et al. 2015; Ono 2015). The main purpose of smooth pursuit eye movements is to minimize the retinal slip, which is generated from the difference between the eye velocity and the target velocity (Shibata et al. 2005; Zambrano et al. 2010). Once the eye velocity catches up to the target velocity, the retinal slip reduces to zero. In studies of the primate smooth pursuit system, the smooth pursuit gain (SPG), which is defined as the ratio of eye velocity to the target velocity, is often used for evaluating the system performance (Marino et al. 2007; Jansson and Medvedev 2011). Recent experiments in humans and monkeys suggest that with a constant velocity or a sinusoidal target motion, the SPG is almost 1.0 (Robinson et al. 1986). However, due to the time delays in the visual pathways, the high SPG of the smooth pursuit system cannot be achieved merely by visual negative feedback methods.

If the target velocity can be predicted, the visual delays can be reduced or even cancelled (Whittaker and Eaholtz 1982; Wells and Barnes 1998; Fukushima et al. 2002). It has been known for a long time that the smooth pursuit system is able to predict the target motion. The first clear evidence for prediction during the smooth pursuit eye movement came from the studies of tracking of repetitive motions, in which the eye was shown to reverse direction in time with, and sometimes shortly before, the target (Dodge et al. 1930; Westheimer 1954). And after that, the predictive tracking has got more attention. Prediction was attributed to special circuitry that came into play only for periodic motions, allowing the pursuit system to learn, and then generate repetitive oculomotor patterns (Dallos and Jones 1963; Barnes and Asselman 1991).To cancel the visual delays, Robinson et al. (1986) proposed a model working as a feed‐forward control. But the model cannot achieve zero‐delay tracking of sinusoidal. Based on Pavel's proposal, a predictive mechanism contained an adaptive filter was integrated into a model of the human smooth pursuit system (Koken et al. 1996). The model provided a fairly good qualitative and mostly also a fairly good quantitative description of human tracking of the various stimuli. Bardshwa et al. (1997) used a Kalman filter for prediction. A target‐selective adaptive control model that performs zero‐delay tracking was proposed (Bahill and McDonald 1983). The above‐mentioned models assumed prior knowledge of the target dynamics and, thus, they avoided addressing how unknown target motion can be tracked accurately.

Some studies investigated horizontal and vertical tracking of moving targets and the vertical tracking was found to be inferior to horizontal tracking at all age levels (Grönqvist et al. 2006). Infants, at 1 month of age, can exhibit smooth pursuit, but only at the speed of 10°/sec or less and with a low gain (Roucoux et al. 1983). The gain of smooth pursuit improves substantially between 2 and 3 months (von Hofsten and Rosander 1997). At 5 months, this ability approaches that of adults. These studies demonstrate that the primate smooth pursuit develops with experience. Based on this, Zambrano et al. (2010) added a memory‐based internal model that stores the model parameters related to the target dynamics to Shibata's model. After the learning phase, the prediction time decreased significantly. The model had the capability to learn the experienced values of the target velocity for ramp and sinusoidal signals, but only in the case of target dynamics already experienced by the system can add the learning component. A model relies on two Kalman filters: (1): one processing visual information about retinal input; and (2) one maintaining a dynamic internal memory of target motion was developed (Orban de Xivry et al. 2013).

However, all of the aforementioned models can only track constant velocity and sinusoidal target motion. To solve this problem, control theory principles have been used to gain understanding on how the different components of the eye tracking system operate. The eye tracking system is assumed to be described by linear time‐invariant discrete‐time state‐space equations without considering the time delays in the visual pathways (Rivlin et al. 1998; Avni et al. 2008). Therefore, taking predictive, learning and time delays nature into account, an information fusion controller with time delays is proposed in this paper to track arbitrary target trajectories. The main idea of the proposed control method is that ideal control strategy, desired trajectory, and eye system dynamics, are all regarded as measuring information of control strategy.

## Primate Smooth Pursuit Eye Movement

Most of the processing in primate vision is devoted to a very small portion of the field of view called “fovea.” The foveal field of view is hardly 2 degrees in extent (Dithcburn 1973), although even within this region there is considerable variation of visual acuity. The movements of the eyes shift the foveal field allowing us high‐resolution vision wherever it is needed. Eye movements have been divided into two categories: smooth pursuit and saccades (Carpenter 1988). In this paper, we are interested especially in smooth pursuit eye movement.

Smooth pursuit eye movements are effective for tracking slow target trajectories. These movements usually have latency of around 100 msec in the visual pathways. Smooth pursuit eye movement occurs when the eye is tracking a smoothly moving target and appears to keep the target image stabilized with respect to retina (Rivlin et al. 1998).

From a neurophysiological point of view, the middle temporal (MT) area and the medial superior temporal (MST) area seem to be intimately involved in smooth pursuit eye movement. In the primate brain, the neural pathways that mediate smooth pursuit eye movement start in the primary visual cortex (V1) and extend to the MT area that serves as the generic visual motion processor (Thier and IIg 2005). The MST area seems to contain the explicit representation of object motion in world–centered coordinates (IIg et al. 2004). Kawawaki et al. (2006) demonstrated that the MST area is responsible for target dynamics prediction. Cortical eye fields are also involved in smooth pursuit (Tian and Lynch 1996); in particular, the frontal eye field can modulate the gain control (Tanaka and Lisberger 2001, 2002) that determines how strongly pursuit will respond to a given motion stimulus. Gain control works as a link between the visual system and the motor system, therefore, motor learning could concern this stage by altering this link (Chou and Lisberger 2004).The cerebellum seems to play a crucial role in supporting the accuracy and adaption of voluntary eye movements. It uses at least two areas for processing signals relevant to smooth pursuit: the flocculus‐paraflocculus complex and the posterior vermis.

## Information Fusion Controller with Time Delay

### Basic theory of information fusion estimation

Theorem 1 (Wang et al. 2007a,b): Suppose all information about the estimated ***x*** ∈ *R* ^*n*^ can be described as follows:

(1) $${\hat{y}}_{i}=H_{i}x+v_{i},i=1,2,\cdots ,m$$

where ${\hat{y}}_{i}\in R^{m_{i}}$ is the measuring value; $H_{i}\in R^{m_{i}\times n}$ is the information mapping matrix; $v_{i}\in R^{m_{i}}$ is the measuring error; and

(2) $$E\left[v_{i}\right]=0,E\left[v_{i}v_{j}^{T}\right]=\left\{\begin{array}{c} R_{i},i\neq j\\ 0,\;i=j \end{array}\right.$$

with $E\left[v_{i}\right]$ and $E\left[v_{i}v_{j}^{T}\right]$ representing the mean and variance of *v*_*i*_, respectively.

If $\sum_{i=1}^{n}H_{i}^{T}R_{i}^{-1}H_{i}$ is nonsingular, then $\hat{x}$ is an optimal fusion estimate of ***x***. Thus, $\hat{x}$ is expressed as:

(3) $$I\left[\hat{x}|x\right]=\sum_{i=1}^{n}H_{i}^{T}R_{i}^{-1}H_{i}$$

(4) $$\hat{x}={\left\{I\left[\hat{x}|x\right]\right\}}^{-1}\sum_{i=1}^{n}H_{i}^{T}R_{i}^{-1}{\hat{y}}_{i}$$

where $I\left[\hat{x}|x\right]$ denotes the information weight (IW) of $\hat{x}$ about itself.

Theorem 1 has solved the problem of closed‐form expression of linear information fusion estimation. For convenience, the following case is considered.

Case 1: If there exists a unit mapping in *H*_*i*_, *i* = 1, 2, ···, *m*, that is, ${\hat{y}}_{j}=x+v_{j}$, then an explicit expression that is easy to recursively calculate can be obtained:

(5) $$\hat{x}={\hat{y}}_{j}+R_{j}\sum_{i=1,i\neq j}^{m}H_{i}^{T}R_{i}^{-1}\left({\hat{y}}_{i}-H_{i}\hat{x}\right)$$

(6) $$I\left[\hat{x}|x\right]=R_{j}^{-1}+\sum_{i=1,i\neq j}^{m}H_{i}^{T}R_{i}^{-1}H_{i}$$

### Information fusion control with time delays for smooth pursuit eye movement

Considering the time delays in the visual pathways, the equations depicting the eye dynamics can be modified as follows:

(7) $$x\left(k+1\right)=A\left(k\right)x\left(k\right)+B\left(k\right)u\left(k-b\right)$$

(8) $$y\left(k\right)=C\left(k\right)x\left(k\right)$$

where $x\left(k\right)={\left[x\dot{x}\right]}^{T}$ denotes the state vector with *x* being the eye position and $\dot{x}$ being the eye velocity, $u\left(k\right)\in R^{1}$ is the control vector; It should be pointed out that $y\left(k\right)=\dot{x}$ is the output vector; *k* = 1, 2, ···, *k*_*f*_;$A\left(k\right)\in R^{2}\times R^{2}$, $B\left(k\right)\in R^{2}$ and $C\left(k\right)\in R^{1}\times R^{2}$ are the state matrix, the input vector and the output vector, respectively; *b* is the length of control lag; $x\left(0\right)={\left[00\right]}^{T}$, $u\left(\tau \right)=0$, $\tau \in \left[-hb,0\right)$, *h* is the sampling period.

Supposing the desired output of the system as $y^{}*\left(k\right)$. Our objective is to control the eye system (1)–(2) in such a way that the output $y\left(k\right)$ tracks the desired output $y^{}*\left(k\right)$ as closely as possible with minimum expenditure of control effort. For this, the performance index is chosen as:

(9) $$J=\|y\left(k_{f}\right)-y^{*}\left(k_{f}\right){\|}_{S}^{2}+\sum_{k=0}^{k_{f}-1}\|y\left(k\right)-y^{*}\left(k\right){\|}_{M}^{2}+\sum_{k=0}^{k_{f}-1}\|u\left(k\right){\|}_{N}^{2}$$

where the first and the second terms on the right‐hand side of (9) represent that the system should track the desired outputs $y^{*}\left(k\right)$, $S\left(k\right)$ and $M\left(k\right)$ denote its IW; the third term denotes the requirement of minimizing the controlled quantity, $N\left(k\right)$ denotes its IW; *k*_*f*_ represents the terminal time; $S\left(k\right)$, $M\left(k\right)$ and $N\left(k\right)$ are positive definite symmetric matrices.

From (8) and (9), it can be obtained that:

(10) $$y^{*}\left(k\right)=y\left(k\right)+m\left(k\right)=C\left(k\right)x\left(k\right)+m\left(k\right)$$

with $m\left(k\right)$ representing a white noise with zero mean and variation of $M^{-1}\left(k\right)$.

It can be obtained from (9) that:

(11) $$0=u\left(k\right)+n\left(k\right)$$

where $n\left(k\right)$ denotes a white noise with zero mean and variation of $N^{-1}\left(k\right)$.

From the point of information fusion estimation, the information of the eye control problem can be grouped into three groups:

1. Hard restriction information determined by (7):
  $$x\left(k+1\right)=A\left(k\right)x\left(k\right)+B\left(k\right)u\left(k-b\right),k=1,2,\cdots ,k_{f}$$
2. Tracking information from the desired trajectory $y^{*}\left(k\right)$:
  $$y^{*}\left(k\right)=C\left(k\right)x\left(k\right)+m\left(k\right),k=1,2,\cdots ,k_{f}$$
3. Control restriction information from minimizing $u\left(k\right)$:
  $$0=u\left(k\right)+n\left(k\right),k=0,1,\cdots ,k_{f}-b-1$$

The purpose of information fusion control is to obtain the optimal fusion estimate:

$$\hat{u}\left(k\right),k=0,1,\cdots ,k_{f}-b-1$$

by fusing all information mentioned above.

Suppose that all information with respect to $x\left(k+b+1\right)$ has been fused, its optimal fusion estimate $\hat{x}\left(k+b+1\right)$ and IW $P\left(k+b+1\right)$ have been obtained at the time of *k + b*. It should be pointed out that, only the future information has the impact on the present decision, and the present information has no influence on the future decision. Therefore, all information with respect to $u\left(k\right)$ can be listed as follows:

1. $$x\left(k+b+1\right)=A\left(k+b\right)x\left(k+b\right)+B\left(k+b\right)u\left(k\right)$$
2. $$0=u\left(k\right)+n\left(k\right)$$
3. $$\hat{x}\left(k+b+1\right)=x\left(k+b+1\right)+\varphi \left(k+b+1\right)$$

where $\varphi \left(k+b+1\right)$ is a white noise with zero mean and variation of $P^{-1}\left(k+b+1\right)$.

Substituting information (1) into information (3), it can concluded that:

(12) $$\hat{x}\left(k+b+1\right)=A\left(k+b\right)x\left(k+b\right)+B\left(k+b\right)u\left(k\right)+\varphi \left(k+b+1\right)$$

Thus, according to (5), we can fuse (12) and information (2) yields:

(13) $$I\left[\hat{u}\left(k\right)|u\left(k\right)\right]=N\left(k\right)+B^{T}\left(k+b\right)P\left(k+b+1\right)B\left(k+b\right)$$

Based on theorem 1, it can be obtained that:

(14) $$\begin{array}{cc} & \hat{u}\left(k\right)={\left[N\left(k\right)+B^{T}\left(k+b\right)P\left(k+b+1\right)B\left(k+b\right)\right]}^{-1}B^{T}\left(k+b\right)\times P\left(k+b+1\right)\\ & \times \left[\hat{x}\left(k+b+1\right)-A\left(k+b\right)x\left(k+b\right)\right] \end{array}$$

Subsequently, we will discuss how to obtain $\hat{x}\left(k\right)$ and its IW $P\left(k\right)$ by fusing all information about $x\left(k\right)$. Similarly, suppose that $\hat{x}\left(k+1\right)$ and its IW $P\left(k+1\right)$ have been obtained at the time of *k*. Thus, the information related to $x\left(k\right)$ can be listed as follows:

1. $$x\left(k+b+1\right)=A\left(k+b\right)x\left(k+b\right)+B\left(k+b\right)u\left(k\right)$$
2. $$0=u\left(k\right)+n\left(k\right)$$
3. $$\hat{x}\left(k+1\right)=x\left(k+1\right)+\varphi \left(k+1\right)$$
4. $$y^{*}\left(k\right)=C\left(k\right)x\left(k\right)+m\left(k\right)$$

Substituting information (2) and (3) into information (1), one has the following:

(15) $$\hat{x}\left(k+1\right)=A\left(k\right)x\left(k\right)-B\left(k\right)n\left(k-b\right)+\varphi \left(k+1\right)$$

with $\varphi \left(k+1\right)$being a white noise with zero mean and variation of $P^{-1}\left(k+1\right)$.(15) can be written as follows:

(16) $$\hat{x}\left(k+1\right)=A\left(k\right)x\left(k\right)+q\left(k\right)$$

where $q\left(k\right)$ represents a white noise with zero mean and variation of $Q^{-1}\left(k\right)$, which is of the following form:

(17) $$Q^{-1}\left(k\right)=P^{-1}\left(k+1\right)+B\left(k\right)N^{-1}\left(k-b\right)B^{T}\left(k\right)$$

Based on theorem 1, by fusing (16) and information (4), one has:

(18) $$P\left(k\right)=A^{T}\left(k\right)Q\left(k\right)A\left(k\right)+C^{T}\left(k\right)M\left(k\right)C\left(k\right)$$

(19) $$\hat{x}\left(k\right)=P^{-1}\left(k\right)\left[A^{T}\left(k\right)Q\left(k\right)\hat{x}\left(k+1\right)+C^{T}\left(k\right)M\left(k\right)y^{*}\left(k\right)\right]$$

The boundary conditions for (18) and (19) are obtained as follows:

(20) $$\hat{x}\left(k_{f}\right)=P^{-1}\left(k_{f}\right)C^{T}\left(k_{f}\right)S\left(k_{f}\right)y^{*}\left(k_{f}\right)$$

(21) $$P\left(k_{f}\right)=I+C^{T}\left(k_{f}\right)S\left(k_{f}\right)C\left(k_{f}\right)$$

### Solution of the eye control problem

Step 1: Set:

(22) $$x\left(0\right)=x_{0}\;\text{and}\;\hat{u}\left(i\right)=0,i=-b,-b+1,\cdots ,-1.$$

Step 2: Compute:

(23) $$P\left(k_{f}\right)=I+C^{T}\left(k_{f}\right)S\left(k_{f}\right)C\left(k_{f}\right)$$

(24) $$\hat{x}\left(k_{f}\right)=P^{-1}\left(k_{f}\right)C^{T}\left(k_{f}\right)S\left(k_{f}\right)y^{*}\left(k_{f}\right)$$

Step 3: Compute:

(25) $$Q^{-1}\left(k\right)=P^{-1}\left(k+1\right)+B\left(k\right)N^{-1}\left(k-b\right)B^{T}\left(k\right)$$

(26) $$P\left(k\right)=A^{T}\left(k\right)Q\left(k\right)A\left(k\right)+C^{T}\left(k\right)M\left(k\right)C\left(k\right)$$

(27) $$\hat{x}\left(k\right)=P^{-1}\left(k\right)\left[A^{T}\left(k\right)Q\left(k\right)\hat{x}\left(k+1\right)+C^{T}\left(k\right)M\left(k\right)y^{*}\left(k\right)\right]$$

(28) $$k=k_{f}-1,\cdots ,1$$

Step 4: Compute:

(29) $$\begin{array}{cc} & \hat{u}\left(k\right)={\left[N\left(k\right)+B^{T}\left(k+b\right)P\left(k+b+1\right)B\left(k+b\right)\right]}^{-1}B^{T}\left(k+b\right)\times P\left(k+b+1\right)\\ & \left[\hat{x}\left(k+b+1\right)-A\left(k+b\right)x\left(k+b\right)\right] \end{array}$$

(30) $$x\left(k+1\right)=A\left(k\right)x\left(k\right)+B\left(k\right)u\left(k-b\right)$$

(31) $$k=0,\cdots ,k_{f}-b-1$$

**Figure 1:**
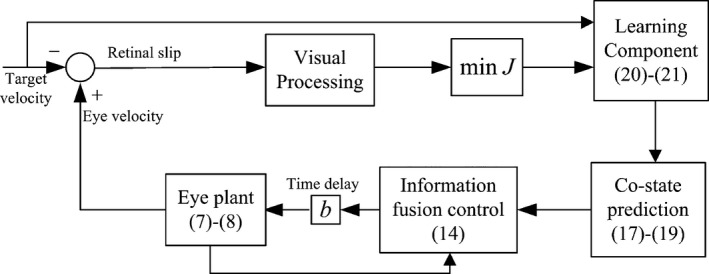
Block scheme of the proposed information fusion control method.

Figure 1 shows a block scheme of the proposed control method of the smooth pursuit eye movement.

## Simulation Results

In order to verify that our control method can achieve smooth pursuit with gain one and zero‐latency, two groups of numerical simulation tests are included. More precisely, in the first group, our control method is compared with Zambrano's model (Zambrano et al. 2010). Then, in the second group, its tracking performance for other target trajectories of eyes is illustrated.

Using Matlab/Simulink, the simulation model is built as a discrete system. According to the spectrum of values reported in the neurobiological literature, the time delay is set to 100 msec. The sampling period is determined as 0.01 sec. The parameters of the eye plant are set as:

(32) $$\begin{array}{cc} & A=\left[\begin{array}{cc} 1 & 9.96\times 10^{-4}\\ 0 & 9.91\times 10^{-1} \end{array}\right],B=\left[\begin{array}{c} 4.74\times 10^{-6}\\ 9.46\times 10^{-3} \end{array}\right],C=\left[\begin{array}{cc} 0 & 1 \end{array}\right]\\ & x\left(0\right)={\left[\begin{array}{cc} 0 & 0 \end{array}\right]}^{T},S=M=10^{4},N=1,h=0.01,b=10,k_{f}=550 \end{array}$$

### Simulation group 1

In this group, we intend to test the presented method's superior tracking performance in comparison with the Zambrano's model. Towards this end, the following two desired trajectories of eyes are considered.

Case 1: sinusoidal motion

(33) $$y^{*}=0.2\pi \sin\left(\pi hk\right)$$

Case 2: constant motion

(34) $$y^{*}=0.6$$

**Figure 2:**
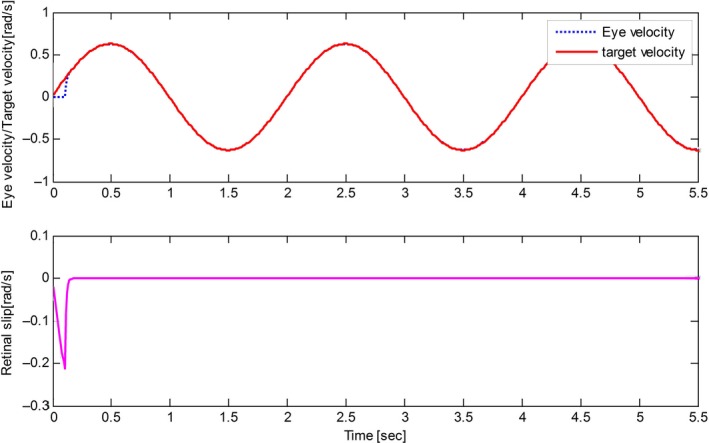
Results of the proposed information fusion control method in the case of a sinusoidal motion of the target.

**Figure 3:**
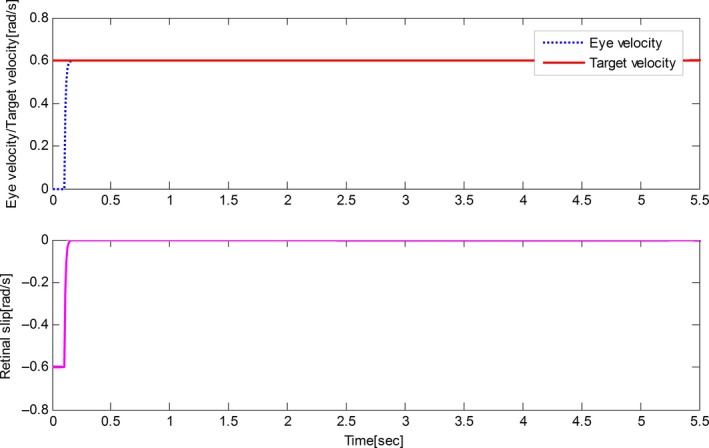
Results of the proposed information fusion control method in the case of a constant velocity motion of the target.

**Figure 4:**
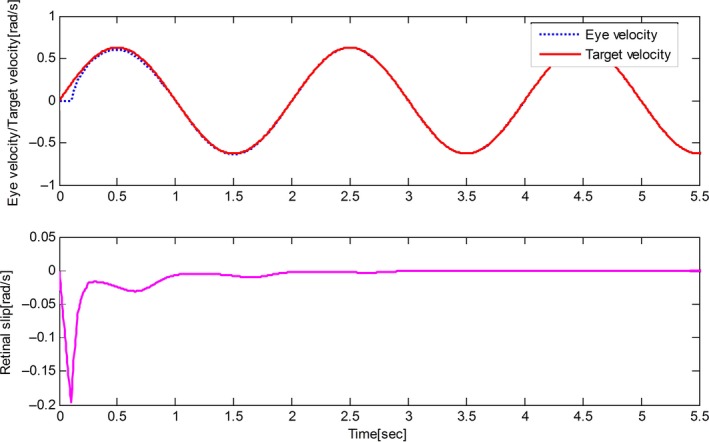
Results of the Zambrano's model in the case of a sinusoidal motion of the target.

**Figure 5:**
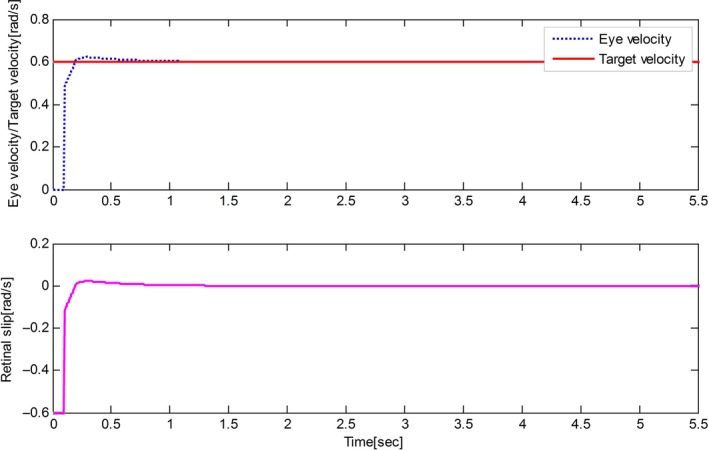
Results of the Zambrano's model in the case of a constant velocity motion of the target.

The derived results are recorded in Figures 2, 3, 4, 5. It is seen that, for both the two cases, both the proposed control method and Zambrano's model achieve excellent steady state tracking result. As can be seen from Figures 2b–5b, for the proposed control method, the retinal slip reaches steady state after 0.1 sec, while more than 0.5 sec for the Zambrano's model. In other words, with the same implementation conditions, the convergence time of the proposed control method is less than that of the Zambrano's model.

### Simulation group 2

Next, the tracking performance of the designed control method for any other desired trajectories of eyes is validated. To do so, the following three desired trajectories are considered

$$\text{Case}\;1:y^{*}=0.1hk;$$

$$\text{Case}\;2:y^{*}=0.2\pi \sin\left(\pi hk\right)e^{-Tk};$$

$$\text{Case}\;3:y^{*}=0.2\pi \cos\left(\pi hk\right)e^{-Tk}.$$

**Figure 6:**
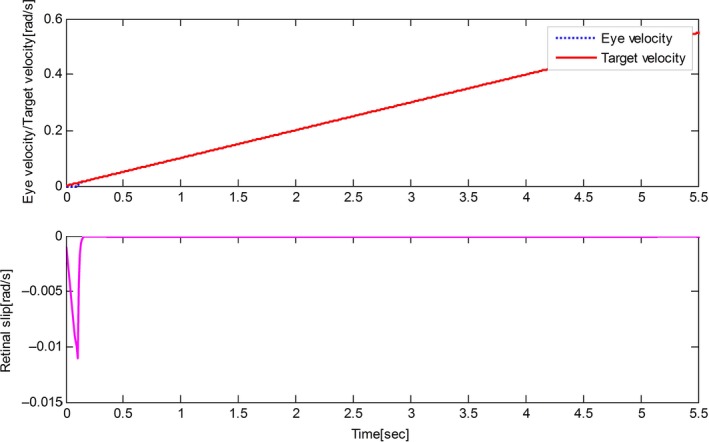
Results of the proposed information fusion control method with respect to case 1.

**Figure 7:**
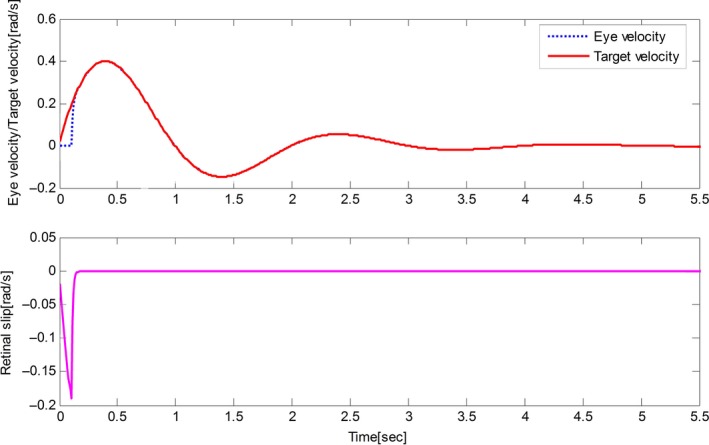
Results of the proposed information fusion control method with respect to case 2.

**Figure 8:**
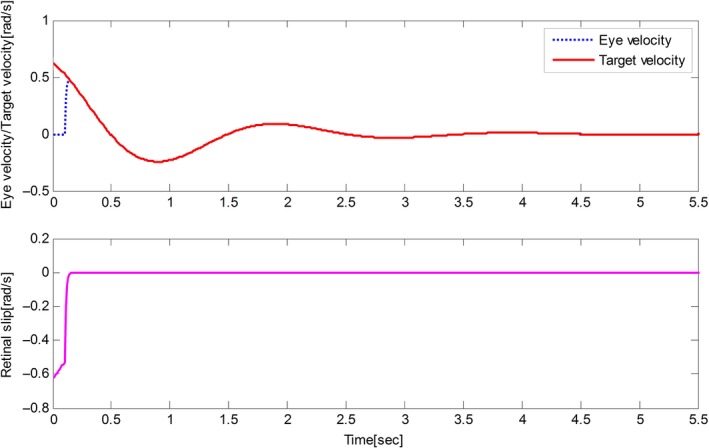
Results of the proposed information fusion control method with respect to case 3.

Figures 6, 7, 8 depict the behavior of the proposed control method for different desired trajectories of eyes. From these figures, it can be seen that the tracking performance, including the tracking efficiency and the convergence time, is not degraded obviously by the change of desired trajectories. This merit brings much convenience for the application of the designed control method in practical eye movement, because the eyes need to track arbitrary trajectories in the practical application.

## Discussion

Information fusion control method elegantly describes in a function manner how the brain may deal with arbitrary target velocities, how it implements the smooth pursuit eye movement with prediction, learning and time delays. These two principles allowed us to accurately describe visually guided, predictive and learning smooth pursuit dynamics observed in a wide variety of tasks within a single theoretical framework.

## Conflict of Interest

None of the authors has conflicts or potential conflicts of interest including relevant financial interests, activities, relationships, and affiliations related to this study.
